# Retrieving nitrogen isotopic signatures from fresh leaf reflectance spectra: disentangling δ^15^N from biochemical and structural leaf properties

**DOI:** 10.3389/fpls.2015.00307

**Published:** 2015-05-01

**Authors:** Christine Hellmann, André Große-Stoltenberg, Verena Lauströ, Jens Oldeland, Christiane Werner

**Affiliations:** ^1^Experimental and Systems Ecology, University of Bielefeld, BielefeldGermany; ^2^AgroEcosystem Research, Bayreuth Center of Ecology and Ecosystem Research, University of Bayreuth, BayreuthGermany; ^3^Institute of Landscape Ecology, University of Münster, MünsterGermany; ^4^Plant Ecology and Conservation, Institute of Botany, Geisenheim University, GeisenheimGermany; ^5^Biodiversity, Evolution and Ecology of Plants, Biocentre Klein Flottbek and Botanical Garden, University of Hamburg, HamburgGermany

**Keywords:** stable isotopes, spectroscopy, δ^15^N, labeling, leaf reflectance spectra, PLS, spatial scale

## Abstract

Linking remote sensing methodology to stable isotope ecology provides a promising approach to study ecological processes from small to large spatial scales. Here, we show that δ^15^N can be detected in fresh leaf reflectance spectra of field samples along a spatial gradient of increasing nitrogen input from an N_2_-fixing invasive species. However, in field data it is unclear whether δ^15^N directly influences leaf reflectance spectra or if the relationship is based on covariation between δ^15^N and foliar nitrogen content or other leaf properties. Using a ^15^N-labeling approach, we experimentally varied δ^15^N independently of any other leaf properties in three plant species across different leaf developmental and physiological states. δ^15^N could successfully be modeled by means of partial least squares (PLSs) regressions, using leaf reflectance spectra as predictor variables. PLS models explained 53–73% of the variation in δ^15^N within species. Several wavelength regions important for predicting δ^15^N were consistent across species and could furthermore be related to known absorption features of N-containing molecular bonds. By eliminating covariation with other leaf properties as an explanation for the relationship between reflectance and δ^15^N, our results demonstrate that ^15^N itself has an inherent effect on leaf reflectance spectra. Thus, our study substantiates the use of spectroscopic measurements to retrieve isotopic signatures for ecological studies and encourages future development. Furthermore, our results highlight the great potential of optical measurements for up-scaling isotope ecology to larger spatial scales.

## Introduction

The ratio of heavy to light stable isotopes is widely used to study ecological processes at different spatial and temporal scales (Dawson et al., 2002; West et al., 2006; Werner et al., 2012). The value of stable isotopes as ecological tracers relies on the fact that their difference in mass does not affect the chemical properties of the element under consideration, but changes the physical behavior, i.e., heavy isotopes usually react slower and build stronger bonds (Fry, 2006). This effect leads to isotopic fractionation during physical changes and chemical reactions, resulting in products that may be isotopically distinct from the substrate. Thus, all kinds of materials have an isotopic imprint that potentially contains information about the origin of its components and the process in which it was formed, providing a natural tracer that has become an important tool for ecologists. Accordingly, measurements of the nitrogen stable isotopic signature, δ^15^N, have been used to disentangle plant nitrogen sources (Handley and Raven, 1992; Hobbie and Hobbie, 2008; Stahl et al., 2011) and δ^15^N has been proposed as an integrator of N cycling through ecosystems (Högberg, 1997; Robinson, 2001; Craine et al., 2009; Temperton et al., 2012; Kleinebecker et al., 2014). The development of the isoscapes concept (from *iso*topic land*scape*) has recently extended the range of applications to include studies of movement across landscapes and spatial pattern in ecosystem functioning (West et al., 2008, 2010; Bowen et al., 2009; Rascher et al., 2012).

Researchers are increasingly aware of the relevance of spatial scales to ecophysiological processes. However, studies realizing an extensive spatial sampling design for ecophysiological measurements are still rare, as limitations of analytical capacity, time and budget often limit sample sizes to a level of either suboptimal spatial resolution or small areal cover (Beever et al., 2006). Thus, new methods are needed to link physiologically informative measures like stable isotopic signatures to techniques which are appropriate for use on different spatial scales.

A suitable technique to up-scale information, e.g., from leaf to landscape level, is optical spectroscopy in the visible and near-infrared (VIS/NIR) spectral range. Measuring the continuous spectrum of radiance reflected by a target at high spectral resolution enables to infer information on its chemical properties. Leaf biochemical constituents exhibit specific absorption features across the visible and NIR regions of the spectrum, which are the result of electron transitions or bond motions induced by electromagnetic radiation of the specific wavelength and are related to the concentration of the respective chemical (Curran, 1989). This relationship has been extensively used to estimate concentrations of various leaf constituents like nitrogenous compounds or supply with other nutrients, pigment concentrations, water content, or structural properties from leaf- to landscape scales, using field spectra, aerial and/or satellite images (see, e.g., reviews by Ustin et al., 2004, 2009; Blackburn, 2007). Examples comprise ecosystem productivity modeling (Martin and Aber, 1997; Ollinger and Smith, 2005), monitoring of plant physiological states (Sims and Gamon, 2002; Dobrowski et al., 2005), diagnosing plant nutritional disorders (e.g., Cu deficiency, van Maarschalkerweerd et al., 2013), ecosystem scale effects of exotic plant invasion (Asner and Vitousek, 2005; Asner et al., 2006) or classification and phylogenetic studies (Asner and Martin, 2011; Asner et al., 2012).

Optical measurements are also well established to measure isotopic compositions of small gas-phase molecules (H_2_O, CH_4_, CO_2_, and N_2_O), using laser absorption spectroscopy in the near- and mid-infrared region, where the bonds in these molecules exhibit rotational–vibrational motions which are strongly influenced by isotopic composition (see, e.g., Kerstel, 2004; Kerstel and Gianfrani, 2008). Moreover, four recent studies by Wang et al. (2007, 2010), Kleinebecker et al. (2009), Elmore and Craine (2011) and Serbin et al. (2014) suggested that δ^15^N of leaves and/or canopies could be modeled using spectroscopic measurements, by calibrating regression models with spectral information as the predictor variables and δ^15^N as the dependent variable. Thus, first results were provided indicating that δ^15^N from field samples can be retrieved using spectral information. However, previous approaches have been unable to determine whether correlations between δ^15^N and spectral information are driven by direct influence of the heavy ^15^N atom on absorption properties of leaf biochemical compounds, or if the effect is based on covariation between foliar δ^15^N and total leaf nitrogen content and/or other leaf properties, like specific leaf area (SLA) and water content, which usually correlate with δ^15^N in field samples (e.g., Wright et al., 2004; Beyschlag et al., 2009; Craine et al., 2009; Elmore and Craine, 2011; Hellmann et al., 2011). Furthermore, if δ^15^N is modeled across different plant species (Wang et al., 2007, 2010; Kleinebecker et al., 2009; Elmore and Craine, 2011), species-specific differences may account for large parts of the total variation in δ^15^N. Thus, there is a need to disentangle whether inherent effects of ^15^N can be derived from leaf spectral signatures when excluding confounding effects of potential covariation with other leaf properties.

In this study, we firstly tested whether δ^15^N and N content can be modeled from fresh leaf reflectance spectra of field samples within a single species, along a spatial gradient of δ^15^N caused by N input from an invasive N_2_-fixing species. Secondly, we designed a controlled ^15^N labeling experiment, where ^15^N content was modified in three plant species without changing foliar nitrogen. We then specifically tested whether different concentrations of the ^15^N isotope can be predicted from fresh leaf reflectance spectra independently of foliar nitrogen content and any other leaf properties.

## Materials and Methods

### Field Measurements

**FIGURE 1:**
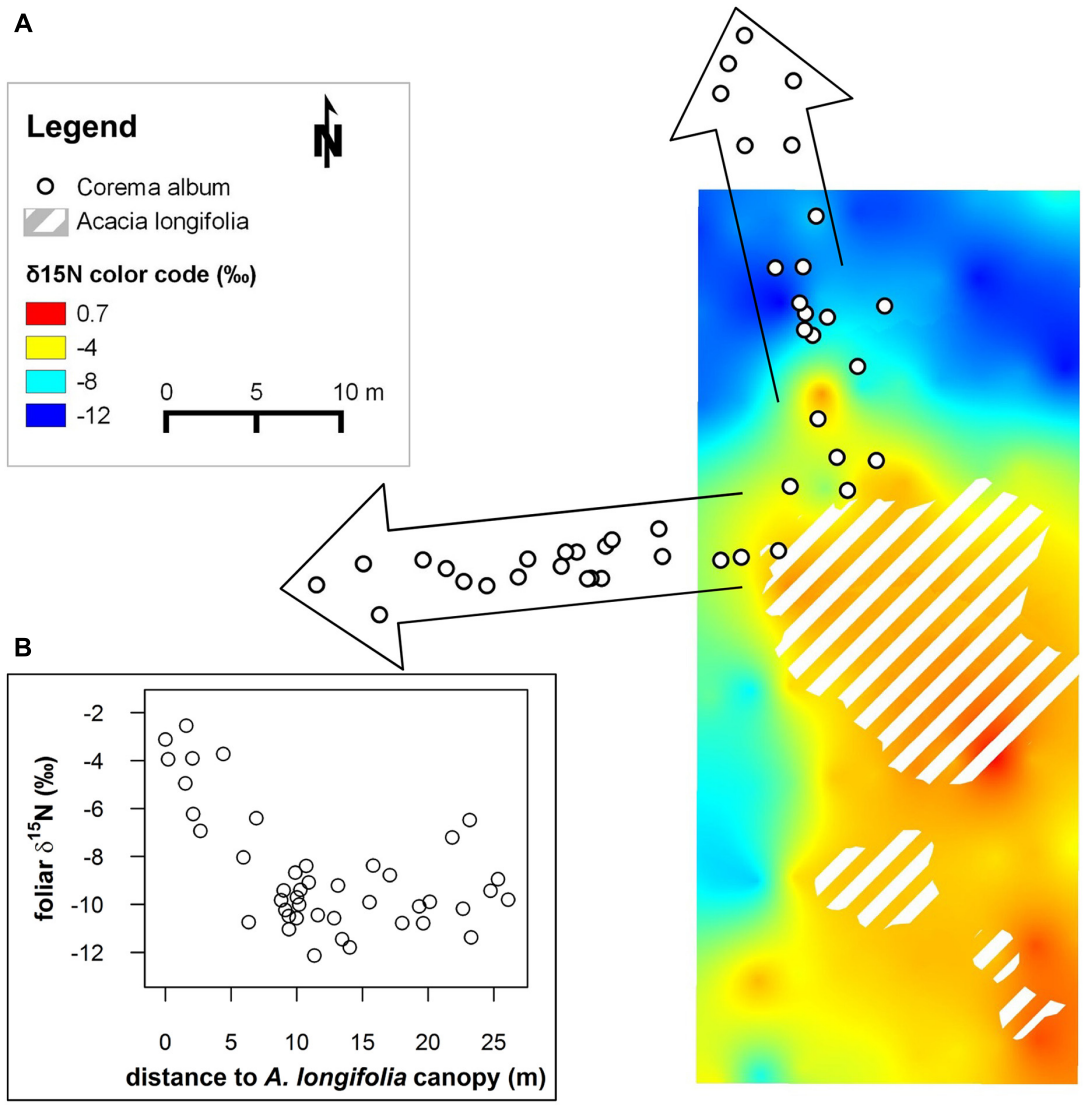
**(A)** ^15^N isoscape depicting δ^15^N values of native, non-fixing *Corema album* interpolated to a continuous surface using ordinary kriging. Canopies of the invasive N_2_-fixing *Acacia longifolia* are demarcated by hatched polygons. δ^15^N is color coded. The change in δ^15^N from strongly depleted values (blue color) to values close to 0‰ (red) in the proximity of the invasive N_2_-fixing *A. longifolia* indicates uptake of fixed N provided by *A. longifolia* (Figure adapted from Rascher et al., 2012; the isoscape was calculated based on leaf δ^15^N values obtained by conventional isotope ratio mass spectrometry). White dots framed by arrows indicate positions of *C. album* individuals along two transects sampled in the current study. Ordinary kriging was performed in SAGA Version 2.1.2 (SAGA Development Team, 2014). **(B)** Inset: foliar δ^15^N (‰) of the transect samples plotted against the distance to the closest *A. longifolia* canopy (m).

Leaf reflectance spectra of *Corema album* L. (Ericaceae) were collected in April 2011 at the primary dunes in Pinheiro da Cruz, Portugal (38°15.39′N, 8°46.31′W; for a detailed site description see Hellmann et al., 2011). Samples were taken at two transects with increasing distance to the canopy of an N_2_-fixing invasive species *Acacia longifolia* (Andrews) Willd (**Figure 1**). Reflectance spectra were recorded using an ASD FieldSpec 3 spectroradiometer equipped with the Plant Probe and Leaf Clip accessory (ASD Inc., Boulder, CO, USA). The ASD Fieldspec 3 has a spectral resolution of 3 nm FWHM (Full-Width Half-Maximum) at 700 nm and 10 nm FWHM at 1400 and 2100 nm. The sampling interval is 1.4 nm for the spectral region 350–1000 and 2 nm for the spectral region 1000–2500 nm and the rate of spectra collection is 10 spectra per second. Spectra average was set to 25 for target measurements and 50 for white reference measurements. The Plant Probe contains a halogen light source and enables contact measurements of individual leaves, thus allowing for high-quality spectra collection under semi-standardized conditions, avoiding effects of changing illumination or atmospheric absorption.

As *C. album* has very small, needle-like leaves of ca. 8 mm length and 1 mm width, which are much smaller than the field of view of the Plant Probe, we pooled a larger amount of leaves per plant for each measurement. Several tips of branches densely foliated with intact current-year’s mature leaves were dissected from the plants and transferred to a transparent plastic bag. Measuring reflectance spectra of foliated twigs inside the plastic bag enabled placing the twigs tightly together and carefully flattening the leaves, so that the twigs were completely covered by leaves and no gaps occurred between leaves. Spectra were referenced against a white 3 mm 0.935 Gortex Leaf Clip Background Disc (ASD Inc, Boulder, Co, USA) covered with the same plastic bag used for the spectral measurement. This referencing procedure successfully removed the effect of the bag in the leaf reflectance spectra, as confirmed by comparing with reflectance spectra of the plastic bag alone (relative to the white reference) and measurements of a pile of dissected leaves taken without plastic bag. After spectral measurements, leaf samples were transferred to paper envelopes and transported to the laboratory, where they were dried at 65°C for at least 48 h and ground to a fine powder with a ball-mill (Retsch, Haan, Germany) for analyses of N content and isotopic composition. As the measured reflectance spectra were clearly dominated by leaves, leaves were dissected from the twigs and used for further analysis. Geographical positions of *A. longifolia* and *C. album* plants were recorded using a differential GPS (GeoHX, GeoExplorer 3000 series, equipped with GA810 Antenna ‘Tornado,’ Trimble, Sunnyvale, CA, USA; accuracy ± 0.6 m, PDOP 2.3, real-time satellite based corrected) and the distance from each *C. album* plant to the canopy of *A. longifolia* was calculated.

### Greenhouse Experiment

Since *C. album* could not be germinated successfully in the laboratory, experiments were conducted with three different Mediterranean species, *Halimium halimifolium* (L.) Willk. (Cistaceae), *Hibiscus rosa-sinensis* L. (Malvaceae), and *Arbutus unedo* L. (Ericaceae). These species grow in the same environment as *C. album*, but have leaf blades which are more favorable for reflectance measurements. *H. rosa-sinensis* is a shrub of East Asian origin and is cultivated in tropical, subtropical and Mediterranean climates. The mesophyllic leaves are relatively large (up to 10 cm long and 6–7 cm wide) and are covered by a thin cuticle. The sclerophyllous shrub *A. unedo* is native to the Mediterranean region and a typical plant of macchia vegetation and evergreen forest. Leaves are ca. 6 cm long and 2–3 cm wide and possess a thick cuticle. *H. halimifolium* is a semi-deciduous shrub of Mediterranean origin, which is frequent on sandy soils, e.g., in dune habitats. It has small, pubescent leaves of ca. 2 cm length and 1 cm width. Mature plants of the three species were grown on sandy substrate and cultivated under controlled conditions in a greenhouse at the University of Bielefeld, Germany, at 22°C air temperature and 60% relative humidity with a light/dark period of 14/10 h and PAR of 551 ± 100 μmol m^-2^s^-1^ (*H. halimifolium*), 457 ± 60 μmol m^-2^s^-1^ (*H. rosa-sinensis*) and 209 ± 57 μmol m^-2^s^-1^ (*A. unedo*), respectively. Plants were rotated weekly. As plants of the same species were obtained from the same cultivation badges and, within species, received same amounts of fertilizer, biomass production, size, growth stage and phenology were fully comparable between individuals. Minimum number of plants per species was *n* = 11 (*A. unedo*), and more plants were used if available (*H. halimifolium*: *n* = 16, *H. rosa-sinensis*: *n* = 12). Plants were then randomly allocated to three treatment levels per species, resulting in 4–6 plants per treatment and species. Treatments were induced by applying fertilizer solution enriched with ^15^N to different degrees (0, 10, and 20‰ enrichment, respectively), while the supply of other elements than ^15^N was exactly the same for all treatments. Because of the lower sample size, *A. unedo* individuals were allocated to the 0 and 20‰ treatments only. Labeled fertilizer solutions were prepared with modified Hoagland’s solution (Peperkorn et al., 2005, with doubled amount of Fe^3+^) by adding 0.274 μmol 98% ^15^N KNO_3_ and 0.145 μmol 95% ^15^N (NH_4_)_2_SO_4_ (Chemotrade, Leipzig, Germany) per 1000 ml Hoagland’s solution and per 10‰ enrichment. Fertilizer solution was obtained from the same stock solution for all treatments, thus ensuring that supply with all elements was equal across treatments. Plants were fertilized once (November 2012) or twice a week (December–February 2013) for 13 weeks with 100-300 ml of the prepared Hoagland’s solution, depending on plant size. All individuals of the same species received equal amounts of fertilizer across treatments, to ensure fully comparable conditions with the only difference between treatments being the different concentration of ^15^N. *H. halimifolium* received solution diluted 1:1 with deionized water, as experience showed that this species is sensitive to high concentrations of nutrients. On all remaining days, plants were watered according to their demand. Plants were maintained under these controlled conditions prior to and throughout the whole experiment. After 13 weeks, total amounts of ^15^N ranging between ca. 1.12 μmol (*H. halimifolium*, 10‰ treatment) to 5.46 μmol (*A. unedo*, 20‰ treatment) had been added per plant.

Spectral measurements were performed in the greenhouse with a FieldSpec 3 spectroradiometer (ASD Inc., Boulder, CO, USA). Spectrum average was set to 30. Leaf reflectance spectra were measured using the FluoWAT leaf clip (Alonso et al., 2007), but without the short-pass filter. An external halogen light source (Osram Decostar 51 Titan 20 W 12 V 36°, Osram GmbH, Munich, Germany) was attached to the FluoWAT such that the lamp would shine directly into the opening at the front. Spectra were referenced against a 1 mm ODM98 foil material (Gigahertz-Optik GmbH, Türkenfeld, Germany) to obtain relative reflectance spectra. Please note that for the field and laboratory measurements, different reference materials and contact probes were used. While the light source of the Plant Probe is perpendicular to the leaf surface and the fiber-optic is mounted at 42° to perpendicular (Serbin et al., 2014), the alignment is other way round in the FluoWAT, with the fiber mounted perpendicular and the light source orientated at 45° to perpendicular (Van Wittenberghe et al., 2013). This may have significant effects on specular reflectance and therefore, no direct comparisons were made between the two data sets.

For spectral measurements in the greenhouse, leaves were detached from the plant, transferred to the leaf clip and spectra were recorded without delay. From each plant, three young and three mature leaves were measured on 5 February 2013. Plants were then subjected to a drought treatment, to test whether modeling would be robust across different physiological states of the leaves. Plants were not watered on three (*H. rosa-sinensis*, *A. unedo*) or four (*H. halimifolium*) consecutive days, which resulted in a marked decline in xylem water potentials by 1.75 ± 0.2 MPa, 1.88 ± 0.5 MPa and 1.37 ± 0.67 MPa, respectively, (mean ± SD) compared to the well watered situation. Three young drought stressed leaves per plant were measured spectrally on 16–17 February 2013.

Each leaf was sealed in a pre-weighted aluminum envelope directly after the spectral measurement and fresh weight was determined. Leaf area was measured using an Image Analysis System (Delta-T Devices Ltd, Cambridge, UK). Leaves were oven-dried at 65°C for 48 h and dry weight was assessed. Gravimetric water content (GWC) and SLA were calculated on a dry weight basis.

### N Content and Isotope Analyses

Leaf samples from the field and the greenhouse experiment were ground to a fine powder and analyzed for nitrogen content and δ^15^N in an Elemental Analyser (HEKAtech GmbH, Weinberg, Germany) interfaced to a continuous flow stable isotope ratio mass spectrometer (ISOPRIME, Elementar, Hanau, Germany). Calibration was conducted using certified standards for N isotopic composition (IAEA-N1, IAEA-N2, International Atomic Energy Agency, Vienna, Austria) and a laboratory standard certified for N content and N isotopic composition (IVA33802156, IVA Analysetechnik e.K., Meerbusch, Germany). To control for potential drift of the instrument, at least two measurements of the laboratory standard were conducted after at least every 10–14 measurements of leaf samples. Isotopic values are expressed in δ notation referenced to the international IAEA standard (AIR). Repeated measurement precision was 0.2‰ for isotope analysis and 0.05% for N content.

### Statistical Analyses

δ^15^N and N content of samples from the labeling experiment were tested for significant differences between treatments and leaf types using a Kruskal–Wallis rank sum test followed by a *post hoc* multiple comparison test (function *kruskalmc* from the R package ‘pgirmess,’ Giraudoux, 2013). Spearman’s rank correlation between foliar δ^15^N values and SLA, GWC and N content was calculated within species and tested for significance (R Version 3.0.1, R Development Core Team, 2013).

### Spectral Data Pre-Processing

The relative reflectance spectra showed characteristic ‘jumps’ at the wavelengths where the ASD instrument switches between different sensors. In all samples, spectral jumps were corrected by applying a multiplicative correction as described in Dorigo et al. (2006), using the SWIR1 sensor as reference. Reflectance spectra were centered on zero and scaled to 1 SD [standard normal variate (SNV) transformation], to reduce scatter effects. Subsequently, the first derivative was calculated to correct for baseline effects and to enhance the resolution of overlapping features. First derivative spectra were calculated with Savitzky–Golay differentiation applying a smoothing window of four points on each side (filter width = nine points) and using the second polynomial. However, the degree of noise in the SWIR2 region of the samples from the labeling experiment was unacceptably high due to a low energy level of the Osram halogen light source in this region. Thus, the wavelength range of 1801–2500 nm was omitted from further analyses. Furthermore, since the mature leaves of *A. unedo* had not incorporated ^15^N label (**Figure 3C**), all 33 mature leaf samples of *A. unedo* had to be excluded from the analyses, in order to avoid a correlation between leaf age and δ^15^N.

### PLS Regressions

Partial least square (PLS) regressions were applied to predict foliar δ^15^N values and, in case of the field samples, N content, from leaf reflectance spectra. PLS regression is a standard method in chemometrics and particularly in model calibration with spectral data, as it was specifically developed for multiple regression problems with many, noisy and collinear variables (Wold et al., 1983, 2001). Similar to PCA, the X matrix is modeled by the product of two smaller matrices, the scores and loadings. In PLS regressions, both, X and Y matrices are modeled simultaneously and the loading matrices are calculated such that the X and Y residuals are small while at the same time the correlation between X and Y scores is maximized (Wold et al., 1983, 2001). In this study, wavelengths ranging from 400 to 1800 nm were included. X and Y data were mean centered and weighted by 1/SD. Non-linear iterative partial least squares (NIPALS) algorithm was used (Wold, 1966, 1975). Due to the limited number of replicates (n = 66 for *A. unedo* after removal of mature leaves) an internal validation using 10-fold cross-validation with random segments was applied as a validation method, as recommended by Kuhn and Johnson (2013). For cross-validation, the dataset is divided into k segments and the model is run k times, with each segment being left out from model calibration in one run and used for validation in this run. This allows for evaluating model performance without ‘losing’ samples for model fit, as well as for significance testing of the regression coefficients, as a set of coefficients is calculated for each submodel and their variation can hence be estimated. This information can be used to select important variables and thus to enhance predictive ability of the model (Marten’s Uncertainty Test, Martens and Martens, 2000).

Outliers were removed from the dataset if they were considered influential outliers in the sense that the sample would strongly influence the model but was not well described by it and probably not representative for the dataset, due to, e.g., measurement or sampling error (for detailed information on outlier removal see Supplementary Table S1).

We applied Marten’s Uncertainty test (Martens and Martens, 2000, see above), in order to eliminate non-informative wavelengths. This procedure was iterated until the predictive ability of the model could not be further improved by the variable selection. More parsimonious models, i.e., models using a small number of factors and/or predicting variables, were generally favored. The number of factors to be used was determined as the first minimum of the root mean square error (RMSE) of prediction. Data pre-treatment and PLS regressions were performed using the software package The Unscrambler X 10.3 (CAMO Software AS, Oslo, Norway).

For the variables used in the final models, variable importance in the projection (VIP) values were calculated as described in Chong and Jun (2005). VIP values are a weighted sum of squares of the PLS-weights with the weights calculated from the amount of Y-variance of each PLS component (Wold et al., 2001).

In addition to the species specific models, a model across all species was built, following the same procedure of spectral pre-treatment (jump correction, SNV transformation, calculation of first derivative and smoothing) and PLS regression with cross-validation and variable selection. For this model, the data was divided into a training- and an independent test set by randomly assigning 2/3 of the samples to the training- and 1/3 to the test set.

## Results

### Field Data

**FIGURE 2:**
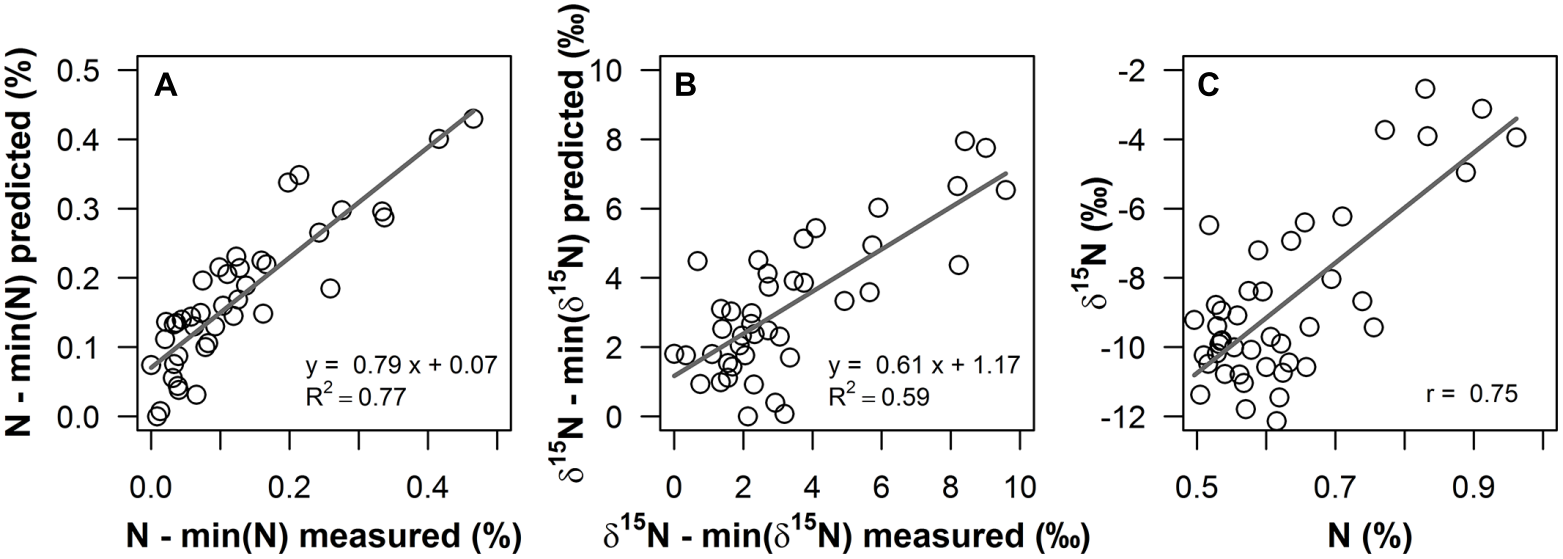
**Measured values versus predicted values from PLS regressions for **(A)** foliar N content (%) and **(B)** δ^15^N (‰) of *Corema album* field samples, using field reflectance spectra in the 400–1800 nm range as predictor variables**. Measured and predicted values were normalized by the minimum value, to illustrate enrichment over values unaffected by *Acacia longifolia*. **(C)**: foliar N content (%) of the field samples plotted against foliar δ^15^N (‰). Regression lines are shown as gray solid lines. Linear regression models and coefficient of determination [*R*^2^, for **(A)**, **(B)**] and Pearson’s correlation coefficient [*r*, for **(C)**] are given.

Foliar δ^15^N in *C. album* varied widely from -12 to -2.5‰ along the transects and showed a pronounced spatial pattern (**Figure 1**). Values were extraordinarily ^15^N-depleted far away from the canopy of *A. longifolia* and became increasingly ^15^N-enriched, i.e., closer to 0‰, the signal of atmospheric nitrogen, the closer the plant grew to the N_2_-fixing invader (**Figure 1**). The maximum difference in δ^15^N was 9.6‰ between the most depleted to the most enriched values within a distance of 25 m. N content varied between 0.47 and 0.96% and was correlated with δ^15^N (*r* = 0.75, **Figure 2C**).

Partial least square regressions of fresh leaf reflectance spectra resulted in accurate and reasonably precise prediction models for δ^15^N and N content of *C. album* field samples. *R*^2^ for the correlation between measured and predicted values was 0.62 for δ^15^N, with a RMSE of cross-validation (RMSE_cv_) of 1.56‰, which corresponds to 16.3% of the range in values (**Figure 2B**). In terms of accuracy and precision, this result was close to the quality of the PLS model on N content, with *R*^2^ = 0.78 (**Figure 2A**) and RMSE_cv_ = 0.05%, corresponding to 10.7% of the range in N content.

### Greenhouse Experiment

**FIGURE 3:**
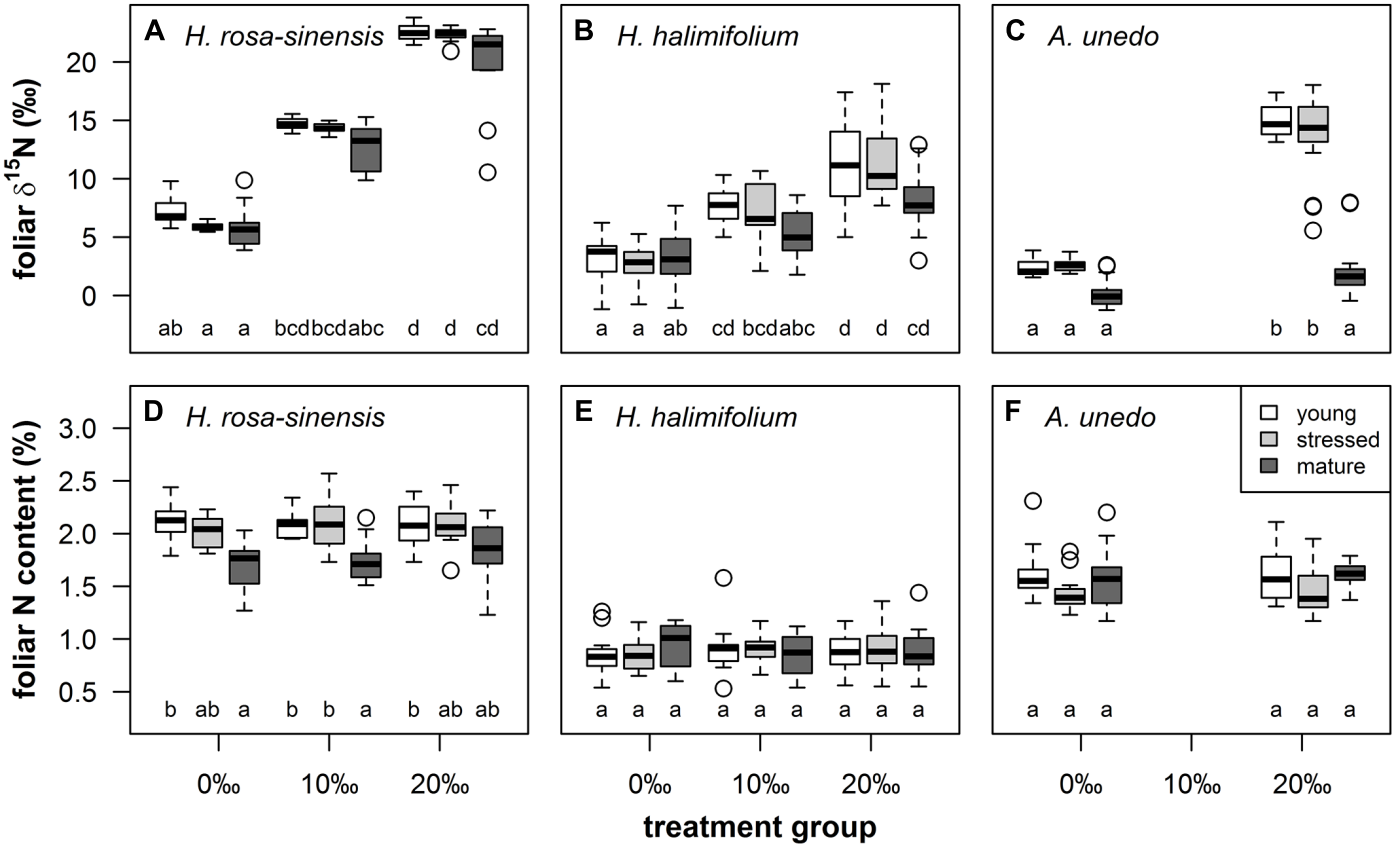
**Boxplots illustrating foliar δ^15^N (‰, first row) and nitrogen content (%, second row) by leaf type (young = white boxes, stressed = light gray boxes, mature = dark gray boxes) and treatment groups (0, 10, and 20‰ enrichment) for *Hibiscus rosa-sinensis* [**(A)**, **(D)**; *n* = 9–12], *Halimium halimifolium* [**(B)**, **(E)**; *n* = 15–18] and *Arbutus unedo* [**(C)**, **(F)**; *n* = 14–18]**. Different lower case letters indicate significant differences at *P* < 0.05 (Kruskal–Wallis test, non-parametric multiple comparisons corrected for α-inflation).

^15^N-labeling generated large gradients in δ^15^N in the treated plants. Total ranges in foliar δ^15^N spanned ca. 16‰ in *A. unedo* and 19‰ in *H. halimifolium* and *H. rosa-sinensis*, respectively (**Figures 3A–C**). For all species and leaf types except mature leaves of *A. unedo*, δ^15^N was significantly enriched in the 20‰ compared to the 0‰ treatment, with the 10‰ treatment ranging in between (**Figures 3A–C**). Generally, mature leaves tended to take up less ^15^N than young and stressed leaves, and labeling failed in mature leaves of *A. unedo* (**Figure 3C**). ^15^N-labeling only affected foliar δ^15^N, while foliar N content did not vary significantly when compared within leaf types and between treatment groups (**Figures 3D–F**), as confirmed by low Spearman’s rank correlation coefficients for N content vs. δ^15^N in all species (0.03 < *r*< 0.2). The correlation between GWC and SLA vs. δ^15^N was similarly weak for *H. rosa-sinensis* and *H. halimifolium* (0.09 < *r*< 0.28). Values were slightly higher for *A. unedo* (*r* = 0.42–0.45), but *R*^2^ was ≤0.2, confirming the independence of foliar δ^15^N from other leaf properties (GWC, SLA and N content).

### Species-Specific PLSR Models

**FIGURE 4:**
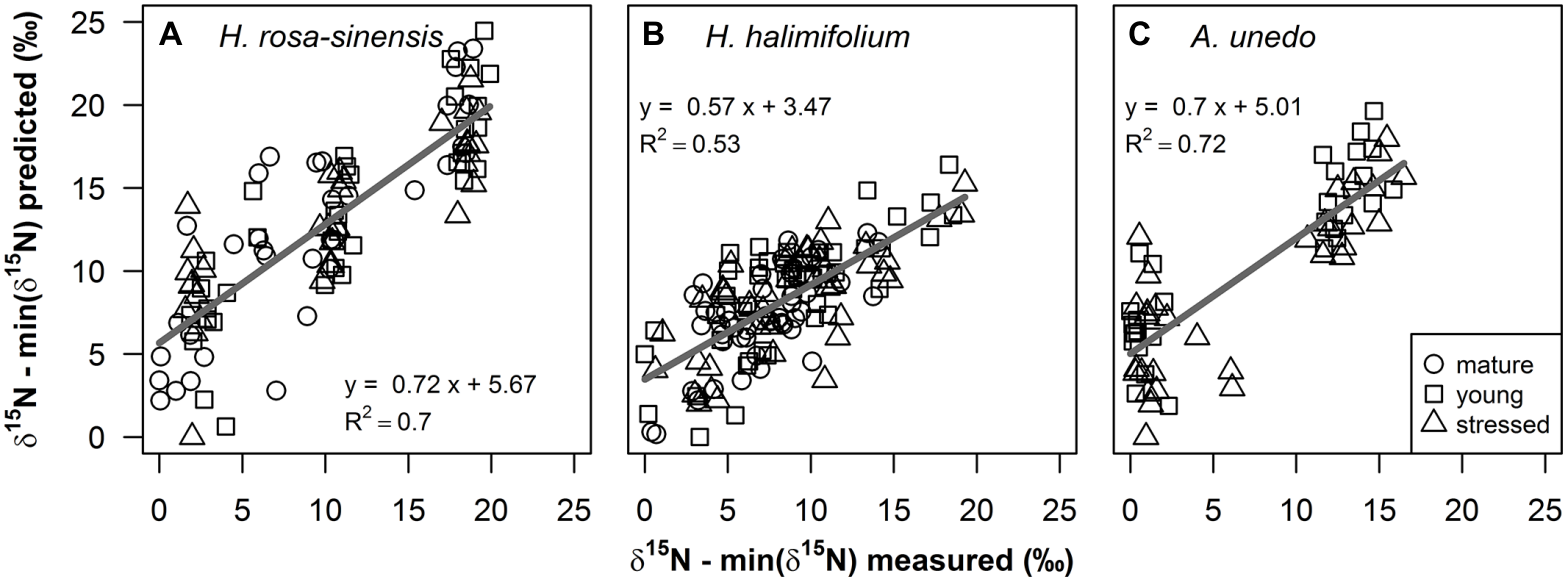
**Measured foliar δ^15^N values versus predicted values (‰) from partial least squares (PLS) regressions on fresh leaf reflectance spectra for **(A)***Hibiscus rosa-sinensis*, **(B)***Halimium halimifolium* and **(C)***Arbutus unedo* are shown for mature (circles), young (squares) and stressed (triangles) leaves**. Measured and predicted values were normalized by the minimum value, to illustrate enrichment over the 0‰ treatment. Regression lines are shown as gray solid lines. Linear regression models and coefficients of determination are given in the figure and additional statistical information is provided in **Table 1**.

δ^15^N of the study plants could be successfully modeled from fresh leaf reflectance spectra using PLS regressions (**Figure 4**; **Table 1**). Predictive ability of the models was high for *H. rosa-sinensis* and *A. unedo*, with cross-validated *R*^2^ of 0.72 and 0.73, respectively, while for *H. halimifolium R*^2^ was lower, but the model still accounted for 53% of the variation in δ^15^N (**Table 1**). The first two factors of the models explained 46–78% of the variation in calibration samples (Supplementary Figures S1D–F). RMSEs of validation were 3.53, 2.84, and 3.28‰ for *H. rosa-sinensis*, *H. halimifolium*, and *A. unedo*, respectively (**Table 1**), which corresponds to 17.7, 14.7, and 19.9% of the range in δ^15^N.

**Table 1:** Model parameters and statistics of partial least squares (PLS) regression models of fresh leaf reflectance spectra to predict foliar δ^15^N values for the three studied species.

species	*n*	No. factors	No. X-variables	No. iterations (MUT)	Offset_cal_	Offset_cv_	Slope_cal_	Slope_cv_	*R*^2^_cal_	*R*^2^_cv_	RMSE_cal_	RMSE_cv_
*Hibiscus rosa-sinensis*	103	4	40	3	2.98	3.94	0.79	0.71	0.79	0.72	3.00	3.53
*Halimium halimifolium*	139	4	117	2	2.54	3.02	0.64	0.57	0.64	0.53	2.48	2.84
*Arbutus unedo*	62	2	39	2	1.9	2.59	0.78	0.70	0.78	0.73	2.89	3.28

MUT, Marten’s Uncertainty Test. Indices *_cal_ and *_cv_ indicate results for calibration and cross-validation, respectively. Accordingly, RMSE_cal_ and RMSE_cv_ are root mean square error of calibration and cross-validation. Offset, slope and *R*^2^ refer to the linear regression models of measured versus predicted values shown in **Figure 4**. *P* ≤ 0.01 for all regression coefficients and *R*^2^ values.

**FIGURE 5:**
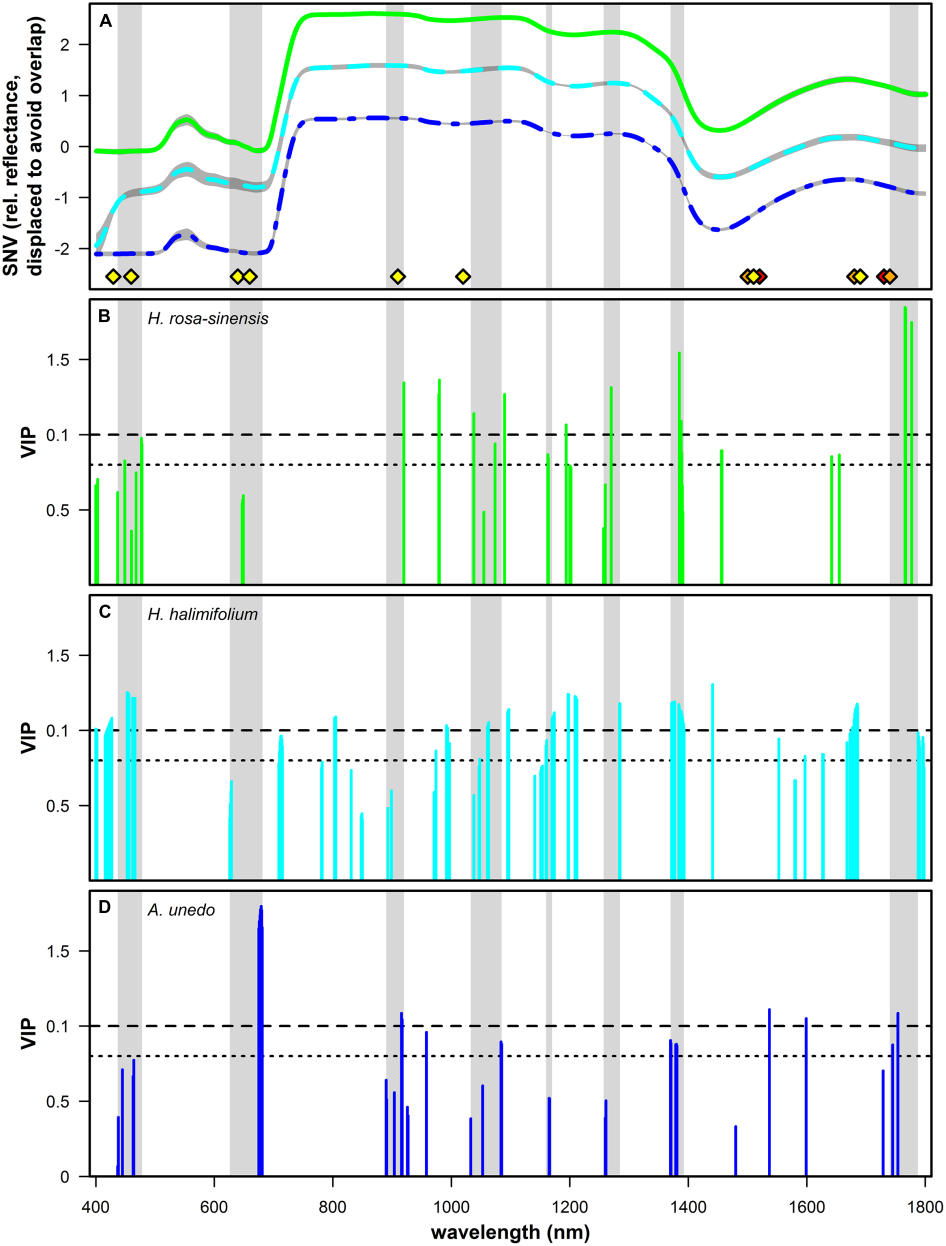
**(A)** Mean of standard normal variate (SNV) transformed relative reflectance spectra of *Hibiscus rosa-sinensis* (green solid line, *n* = 103), *Halimium halimifolium* (cyan dashed line, *n* = 139) and *Arbutus unedo* (dark blue dashed and dotted line, *n* = 62). Gray shaded areas indicate mean ± SD. Spectra of *H. rosa-sinensis* and *A. unedo* were displaced on the *y*-axis by +1 and -1, respectively, to avoid overlap. Absorption features related to N containing bonds taken from literature are shown (Curran, 1989: yellow diamonds; Elvidge, 1990: orange diamonds; Fourty et al., 1996: red diamonds). Light gray bands highlight spectral regions that were consistently significant for predicting δ^15^N across species. **(B–D)**: variable importance in projection (VIP) values are shown for the wavelengths used in PLS regressions and identified as significant with Marten’s Uncertainty Test for *H. rosa-sinensis* (**B**, green), *H. halimifolium* (**C**, cyan) and *A. unedo* (**D**, dark blue). Dashed and dotted horizontal lines indicate values of 1 (mean of all squared VIP values) and 0.8, respectively, which were previously used as a criterion for variable selection (Wold, 1995; Chong and Jun, 2005).

**Figure 5** illustrates the measured reflectance spectra for the three studied species (**Figure 5A**) and the VIP values at spectral bands found to be significant for predicting δ^15^N in this study (**Figures 5B–D**). VIP values are a weighted sum of squares of the PLS-weights for the factors used in the model, with high values indicating high importance. Values of 1, which is the mean of all squared VIP values, and 0.8, respectively, have previously been proposed as criteria for variable selection (Wold, 1995; Chong and Jun, 2005). Important wavelengths were found for all three species in the visible part of the spectrum, at the chlorophyll absorption bands at 416-478 nm and around 626-681 nm, including the red edge in *H. halimifolium*. Further bands which were important for predictions in all species were located in the short wave infrared (SWIR), at 890-920 nm and around 1040, 1165, 1270, and 1375 nm. All species had further important wavelengths in the region between 1400 and 1800 nm; however, these were not consistent between species.

Patterns of VIP values showed some species-specific differences (**Figures 5B–D**). For *H. rosa-sinensis*, wavelengths were more important in the SWIR compared to the VIS region, with the most important bands located at 1385, 1766, 1767, and 1777 nm. For *H. halimifolium*, VIP values were more similar across the spectrum, with important regions at 450-460 nm and around 1200, 1375, and 1680 nm. For *A.unedo*, bands at 678-679 nm were by far the most important to predict δ^15^N, with further important regions located in the SWIR region of the spectrum, at 917, 1537, 1599, and 1754 nm.

### PLSR Model Across Species

The model across all three species performed worse compared to species-specific models, with regression slopes of 0.38, and 0.36, *R*^2^ of 0.38 and 0.35 and RMSE of 5.02 and 5.71 for calibration and validation in the training set, respectively (Supplementary Figure S2B). On factor 1 and 2, PLSR primarily separated the samples into species, and the model performed poor in predicting δ^15^N (Supplementary Figures S2A,B). Prediction accuracy and precision for the test set was weaker compared to the training set, with a slope of 0.2 for the regression of predicted versus measured values, *R*^2^ of 0.28 and RMSE of 5.96‰, which corresponds to 24.58% of the range in δ^15^N values (Supplementary Figure S2E). Samples clustered according to species in the scores plot (Supplementary Figure S2D), but also in the measured versus predicted plot (Supplementary Figure S2E), with no differentiation in predicted δ^15^N values within, but only between species. Accordingly, δ^15^N of *A. unedo* on average was overestimated while values for *H. rosa-sinensis* were underestimated (Supplementary Figure S2F).

## Discussion

### Retrieving δ^15^N from Field Reflectance Spectra

We took advantage of a well-documented gradient in foliar δ^15^N in the native dune shrub *C. album* growing in the surroundings of the N_2_-fixing *A. longifolia*, which is highly invasive in Mediterranean dune systems. In this system, δ^15^N has been proven a sensitive functional tracer for N-input by the invader, allowing to resolve the spatial dimension of changes to the N cycle in the native system by means of isoscapes computation (**Figure 1**, Rascher et al., 2012). ^15^N natural abundance can be used to disentangle plant N sources, provided that the source signals differ markedly (Högberg, 1997; Robinson, 2001). In the studied system, *A. longifolia* is known to heavily enrich the system with nitrogen originating from symbiotic fixation of atmospheric N_2_, which has, by definition, an isotopic signature of 0‰. Thus, the enrichment in foliar δ^15^N in the native, non-fixing *C. album* from the background signal of ca. -12‰ in the native system to the atmospheric value of 0‰ in the vicinity of *A. longifolia* indicates uptake and use of nitrogen which originates from the invader and was provided by N_2_-fixation (Hellmann et al., 2011; Rascher et al., 2012). Here, we aim to evaluate the potential of optical measurements to assess variations of δ^15^N along the spatial gradient, by measuring leaf reflectance spectra in *C. album* along two transects with increasing distance to *A. longifolia* (depicted in **Figure 1**) and calibrating regression models with foliar δ^15^N assessed using standard methods (isotope ratio mass spectrometry).

Indeed, these spatial changes in foliar δ^15^N could be detected in fresh leaf reflectance spectra. δ^15^N was successfully modeled from the leaf reflectance spectra, with *R*^2^ = 0.62 and a reasonable RMSE of cross-validation (RMSE_cv_) of 1.56‰ (**Figure 2B**). This result indicates a similarly good predictability of δ^15^N compared to N content (**Figure 2A**, *R*^2^ = 0.78, RMSE_cv_ = 0.05%). To our knowledge, this is the first study using fresh leaf reflectance spectra to model a field gradient in δ^15^N within one species, thereby demonstrating the potential to spectroscopically quantify the impact of an invasive species, using δ^15^N as a functional tracer. This result furthermore indicates that spectroscopic determination of foliar δ^15^N in the field is potentially a promising cost-effective and rapid tool to resolve the spatial dimension of plant–plant interactions and to study underlying ecological processes, eventually allowing for acquisition of a high number of samples and thus sampling designs with high spatial resolution. Until now, only few studies have attempted to model δ^15^N from reflectance measurements. Kleinebecker et al. (2009) and Serbin et al. (2014), using NIR reflectance spectroscopy of dried and ground leaf samples, presented models with high predictive abilities, ranging between *R*^2^ = 0.6–0.99. Models on fresh leaf reflectance spectra in Wang et al. (2007, 2010) were similarly accurate, with *R*^2^ = 0.83–0.92 across five savannah shrub and grass species. In Elmore and Craine (2011), predictive ability was rather weak for models on VIS/NIR spectra of dried and ground leaves (*R*^2^ = 0.34). However, they performed a relatively conservative pre-selection to three specific absorption features related to nitrogen, cellulose and lignin. The weak predictive ability, compared to the other studies, indicates that further important spectral regions might probably be located across the continuous spectrum. However, all previous studies used field samples, in which covariation of δ^15^N with leaf chemical or structural properties likely occurred. Accordingly, the correlation of δ^15^N with total N content was favored as the most likely mechanism explaining the correlation of δ^15^N with reflectance spectra in Wang et al. (2007, 2010) and similarly, Elmore and Craine (2011) argue that a general, robust relationship between reflectance and δ^15^N was lacking in their leaf-based data probably because of the lack of a correlation between δ^15^N and N content. Likewise, as shown in **Figure 2C**, in the data presented here, foliar N content and δ^15^N are correlated (*r* = 0.75). Thus, at this point and based on measurements of field samples, it was still not clear whether variations in ^15^N content alone alter absorption properties of nitrogen containing chemical bonds, thus leading to detectable changes in the leaf reflectance spectrum. Consequently, experimental evidence was needed to test for a measurable effect of ^15^N on leaf optical properties.

### Experimental Evidence for the Influence of δ^15^N on Leaf Reflectance Spectra

^15^N-labeling effectively induced large variations in δ^15^N in the experimental plants, generating ranges of ca. 16–19‰ (**Figures 3A–C** and **4**). This corresponds to naturally occurring ranges for foliar δ^15^N that can be found across (Beyschlag et al., 2009; Kleinebecker et al., 2009; Temperton et al., 2012) or even within species (**Figures 1** and **2**, Rascher et al., 2012). Furthermore, it was shown that foliar δ^15^N varied independently of GWC, SLA and N content (**Figures 3D–F**) and no covariation with these leaf properties will obscure the interpretation of the PLS models. Hence, the plant material produced by ^15^N labeling proved suitable to separate effects of δ^15^N and other leaf characteristics on leaf reflectance spectra.

Results of the PLS regressions show that δ^15^N of the study plants could be successfully modeled from fresh leaf reflectance spectra with good accuracy and precision (**Figure 4**, **Table 1**). *R*^2^ and RMSE of cross-validation and calibration were close to each other, respectively, confirming the robustness of the models (**Table 1** and Supplementary Figures S1A–C). This shows for the first time that information on foliar δ^15^N can indeed be retrieved from fresh leaf reflectance spectra independently of N content, GWC and SLA within individual species, and thus, that the heavy stable isotope itself has an inherent effect on reflectance spectra.

Significant bands from PLS regressions show reasonable clustering across species, confirming that some spectral regions correlated to foliar δ^15^N are consistent (**Figures 5B–D**). In the visible part of the spectrum, important wavelengths matching the chlorophyll absorption features centered at 430 and 460 nm were found for all three species, and at 640 and 660 nm for *H. halimifolium* and *H. rosa-sinensis* (**Figures 5B–D**; Curran, 1989). Likewise, these regions were suggested by Wang et al. (2007, 2010), who found chlorophyll absorption bands to be most strongly correlated to δ^15^N. They speculate that correlations in these regions arise from variable widths of chlorophyll absorption bands in samples with different ^15^N abundances. Similarly, many of the wavelengths identified here can be related to regions which have been used for nitrogen quantification in previous studies, and thus, may represent absorption features of N containing bonds which are potentially sensitive to isotopic composition. For example, in the NIR, bands at 890–920 nm were selected for all three species (**Figures 5B–D**). This region can be assigned to the C–H stretch from nitrogen-containing proteins (Curran, 1989) and has also been found to be significant in several studies estimating N content of leaves and vegetation (Yoder and Pettigrew-Crosby, 1995; Curran et al., 2001; Mitchell et al., 2012b). Significant bands around 1040 nm presumably correspond to an absorption feature caused by an N-H stretch from protein centered at 1020 nm (Curran, 1989) and were used for nitrogen quantification in Curran et al. (2001) and Mitchell et al. (2012b). Band clusters identified around 1165, 1270, and 1375 nm were consistent with wavelengths used by Min and Lee (2005) and Mitchell et al. (2012a,b), respectively. In the study by Serbin et al. (2014), the bands most important for δ^15^N prediction were located around 1200 nm and at 1450, 1650, 1690, and 1720 nm, matching features in *H. rosa-sinensis* and *H. halimifolium* (1200, 1450, 1650, 1690 nm) and *A. unedo* (1720 nm). In summary, the link of important wavelengths found in this study to absorption features related to nitrogen containing compounds further substantiates the assumption that the concentration of the ^15^N stable isotope directly influences absorption properties of chemical bonds and thus the spectral signatures of leaves.

Apart from the spectral regions mentioned above, wavelengths relevant for δ^15^N prediction are species-specific to some extent, regarding the variability between species in locations as well as importance of significant bands (**Figures 5B–D**). One reason for species specificity could lie in the experimental design, if freshly assimilated labeled N was used differentially in the studied species. For example, the region which is the most important for the prediction in *H. rosa-sinensis* is at 1767–1777 nm. This corresponds to a C–N stretch which was related to an absorption feature of protein, specifically, Rubiso, in Elvidge (1990) and suggests that labeled N assimilated by *H. rosa-sinensis* might have been preferentially used for protein synthesis in the photosynthetic pathway. In contrast, in *A. unedo*, the most important feature was located at the chlorophyll absorption at 675–681 nm, indicating that a larger portion of newly assimilated N might have been incorporated in chlorophylls. In *H. halimifolium*, the chlorophyll absorption features were also significant, but more important in the 453–466 nm range. Nevertheless, in spite of difference in the importance of individual wavelength, several regions were identified that were in common across all species (**Figure 5**).

Species in this experiment were deliberately chosen to represent differing leaf types. Therefore, species specific differences could furthermore be due to leaf structure, leaf surface properties (i.e., specular reflectance) and interactions with other biochemical components, that may have high influence on leaf reflectance spectra and on specific absorption features (Ustin et al., 2004). For instance, trichomes on the leaf surface of *H. halimifolium* may potentially complicate the analysis of leaf biochemicals using fresh leaf spectra (Bousquet et al., 2005; Levizou et al., 2005), and might be one reason for the lower performance of the model in this species. Further experiments, e.g., investigating a greater number of species, systematically testing effects of leaf structure and morphology or varying concentrations of other nutrients as well as including temporal variation, will help to better understand such interactions. Also, including information on intra-plant variation of δ^15^N (Evans, 2001; Gauthier et al., 2013), by applying compound-specific analyses, will enable to establish a better mechanistic link between important wavelength from PLS models and N-related absorption features.

The PLS model across species was also affected by species specificity. Presumably due to pronounced differences, e.g., in leaf structural, chemical and/or physiological properties, PLSR primarily separated the samples into species (Supplementary Figure S2A) and practically failed in predicting δ^15^N (Supplementary Figure S2B). The variation in δ^15^N that is explained by the model seems to relate to differences of mean δ^15^N between the studied species, i.e., higher enrichment in *H. rosa-sinensis* compared to *A. unedo* and *H. halimifolium*, such that species identity to some extent correlated with δ^15^N, and PLS regression is unsuccessful in resolving δ^15^N within species. This becomes even more apparent when evaluating the independent test set, where no differentiation in predicted δ^15^N values appears within, but only between species (Supplementary Figure S2E). In contrast to our results on fresh leaf reflectance spectra, studies using NIRS analysis with dried and ground samples received highly accurate and precise results for models across species (Kleinebecker et al., 2009; Serbin et al., 2014). This indicates that it might indeed either be structural and/or leaf surface properties, including effects of compartmentation, or the presence of water in the samples that hamper combined analysis of strongly differing species, as spectral analysis of dried and ground samples is well known to provide control over these problems (Yoder and Pettigrew-Crosby, 1995). Moreover, in this experiment only three species were included which strongly differed in leaf characteristics, compared to seven species in Kleinebecker et al. (2009) and 46 species in Serbin et al. (2014) and thus, increasing the number of species to be used for model calibration might enhance the ability to model across species.

**FIGURE 6:**
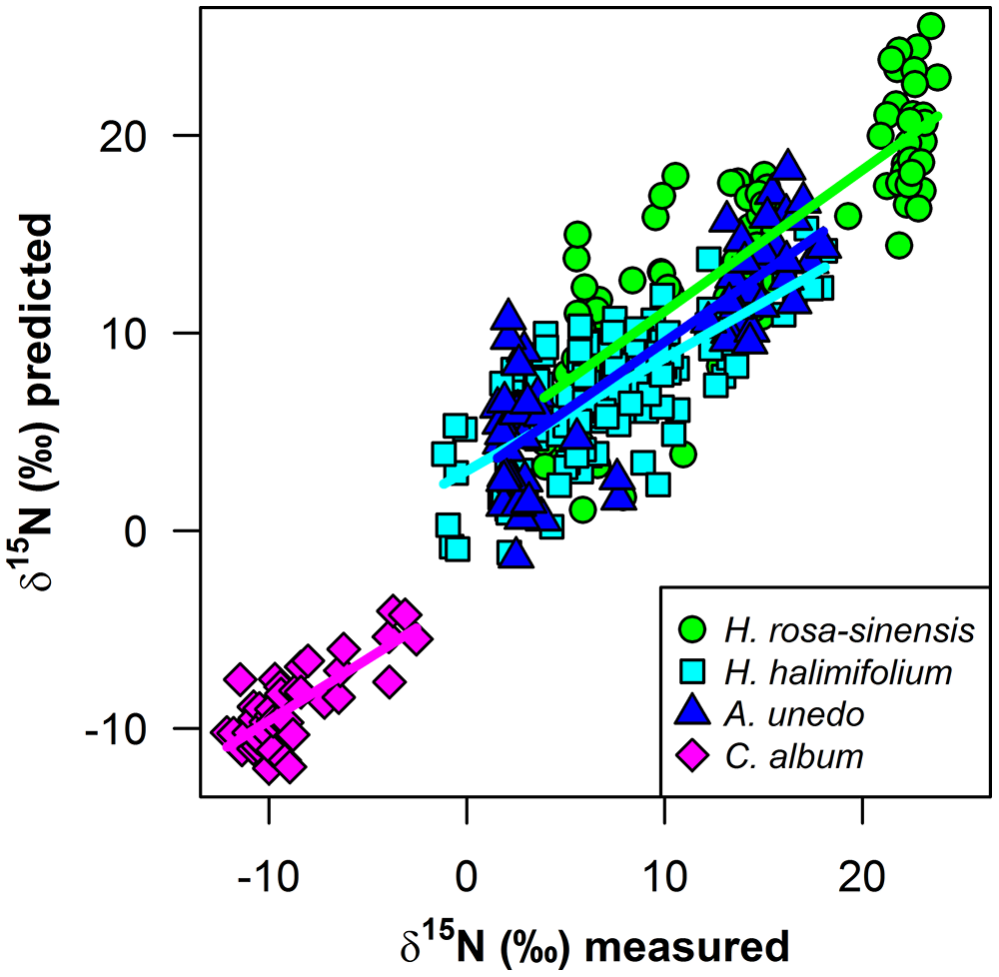
Measured versus predicted foliar δ^15^N (‰*) from PLS regressions for *H. rosa-sinensis* (green circles), *H. halimifolium* (cyan squares), *A. unedo* (dark blue triangles), and *Corema album* field samples (magenta diamonds). Regression lines are shown for each model as solid lines in the matching color for each species

The quality of the models developed in this study is comparable between field and laboratory data across the large range in δ^15^N values (**Figure 6**), yet, the models presented here are still of limited predictive power given the rather high RMSEs, which amount to 15–20% of the range of δ^15^N (**Table 1**), and a bias from the 1:1 line, which leads to a slight underestimation of enriched and overestimation of depleted δ^15^N values in all species. However, by experimentally varying δ^15^N and effectively eliminating covariance with other leaf properties as an explaining factor, our study provides first evidence that there is indeed an effect of the heavy N isotope on leaf reflectance spectra that can be spectroscopically detected and used to predict foliar δ^15^N. With further advance in sensor performance and development of measuring techniques and statistical models, the ability to resolve subtle spectral features such as changes in reflectance induced by substitution of one isotope by another will be enhanced. Therefore, we expect predictions of spectrally measured foliar δ^15^N to improve in the future, and our results clearly encourage further efforts in this field of research. Specifically, including the NIR spectral region between 1800 and 2500 nm may further increase predictive ability, as there is indication that important features are located in this region (Curran, 1989; Elvidge, 1990; Fourty et al., 1996; Serbin et al., 2014). Thus, we suggest that stable isotope research should make use of the great potential of spectroscopic measurements, which may provide an easy, rapid and cost-effective means to produce datasets with a high number of samples, allowing for high spatial resolution and enabling scaling-up isotopic information to large spatial scales. As shown in this study, leaf reflectance spectra can be used to resolve the variation in foliar δ^15^N within one species and could thus effectively trace profound alterations imposed on the native ecosystem by an exotic N_2_-fixing invader along a spatial gradient. Similarly, spatially resolved measurements of δ^15^N in vegetation could be used in further situations where small-scale resolution of plant N sources is of interest, e.g., in studies on competition and facilitation, or wherever an N source with a distinct isotopic signature is affecting surrounding systems, like N input from agriculture, intensive farming or industrial pollution. The significance of the spatial dimension of interactions, alterations, and feedbacks within ecosystems is increasingly acknowledged and the important role of stable isotopes as spatial tracers for ecological processes is reflected in the expanding range of applications of isoscapes (West et al., 2010; Rascher et al., 2012). In Wang et al. (2007, 2010), δ^15^N was modeled using canopy reflectance spectra, further encouraging efforts toward up-scaling spectroscopically assessed isotopic signatures from leaf- to canopy scale and beyond. Recently, in two pioneering studies, empirical relationships between isotopic signatures and spectral indices based on hyperspectral imagery have been established. Santos et al. (2012) showed that δ^13^C correlates negatively with PRI in submerged aquatic plants and Bai et al. (2013) found δ^15^N to correlate negatively with NDVI in grasslands encroached by N_2_-fixing trees. Thus, first evidence for empirical relationships at the community- and landscape scale has already been provided. The knowledge about an inherent effect of ^15^N, which is evident independently of other leaf properties, gives more substance to these results and further highlights the great potential of spectroscopically retrieved isotopic signatures to trace ecological interactions at large spatial scales.

## Author Contributions

CW and CH conceived of and designed the study. JO, AS, and CH collected field data and VL and CH performed the laboratory experiments. CH, VL, and JO analyzed the data, with assistance of CW. CH wrote the first draft of the manuscript and all authors contributed substantially to revisions.

## Conflict of Interest Statement

The reviewer, Till Kleinebecker declares that, despite being affiliated to the same institution as author André Große-Stoltenberg, the review process was handled objectively. The authors declare that the research was conducted in the absence of any commercial or financial relationships that could be construed as a potential conflict of interest.
