# Supplementary material for: Retrieving nitrogen isotopic signatures from fresh leaf reflectance spectra: disentangling δ15N from biochemical and structural leaf properties
Source: Front Plant Sci. 2015 May 1;6:307. doi: 10.3389/fpls.2015.00307 (PMC4416452; doi:10.3389/fpls.2015.00307)
Supplement: Supplementary file 1 [file Image_1.PDF]

Supplementary Material

Retrieving nitrogen isotopic signatures from fresh leaf reflectance spectra: disentangling $\delta^{15}N$ from biochemical and structural leaf properties

Christine Hellmann[1,2,*], André Große-Stoltenberg[3], Verena Lauströer[1,4], Jens Oldeland[5], Christiane Werner[2]

[1]Experimental and Systems Ecology, University of Bielefeld, Bielefeld, Germany
[2]AgroEcosystem Research, University of Bayreuth, BAYCEER, Bayreuth, Germany
[3]Institute of Landscape Ecology, University of Münster, Münster, Germany
[4]Plant Ecology and Conservation, Institute of Botany, Geisenheim University, Geisenheim, Germany
[5]Biodiversity, Evolution and Ecology of Plants, Biocentre Klein Flottbek and Botanical Garden, University of Hamburg, Hamburg, Germany

*** Correspondence:** Christine Hellmann, Experimental and Systems Ecology, University of Bielefeld, Universitätsstraße 25, 33615 Bielefeld, Germany.
christine.hellmann@uni-bielefeld.de

1. Supplementary Data

1.1. Outlier removal

Samples were considered influential outliers and were removed from the dataset if they had high leverage values and at the same time high residual errors, meaning that they had high influence on the model but were not well described by it. Such samples differ from the majority of samples and they tend to influence the model negatively and reduce overall predictive ability, as they describe features which are not common to the majority of samples.

Samples were evaluated by inspecting the influence plots (leverage/ Hotelling T^2 plotted against X or Y) and the X-Y relation (relation between scores of the X and Y matrices) on all factors relevant for the respective model. Samples were removed if they showed high values for Hotelling T^2 above a critical limit (given by an F-test with the significance value set to $p = 0.01$) and at the same time had high Q residuals and/ or were identified as X-Y outliers. Samples with high Hotelling T^2 that were well described by the model remained in the analysis.

2. Supplementary Tables

2.1. Outlier removal

Supplementary Table S1. Samples removed from the analyses, stating reason for removal and probable explanation for distinctness of the samples.

species	sample ID	reason for removal	probable explanation
C. album	24	high leverage, X-Y-relation outlier	bad spectrum
C. album	23	high leverage with high residual X variance	bad spectrum
A. unedo	89	extremely high X residual	bad spectrum
A. unedo	15, 17	high leverage with high residual X-variance; X-Y-relation outlier	particularly young leaves
A. unedo	7	high leverage with high residual Y-variance; X-Y-relation outlier	particularly young leaf
H. rosa-sinensis	62	multivariate (X) outlier; X-Y-relation outlier	bad spectrum
H. rosa-sinensis	53	multivariate (X) outlier; X-Y-relation outlier	bad spectrum
H. rosa-sinensis	90, 100	high leverage with high residual Y-variance; X-Y-relation outlier	water stressed leaves, probably dying
H. rosa-sinensis	22	multivariate (X) outlier	unclear
H. halimifolium	140	high leverage with high residual Y-variance	bad spectrum
H. halimifolium	61	high leverage with high residual Y-variance, X-Y-relation outlier	bad spectrum
H. halimifolium	29	high X residual	bad spectrum; uneven leaf
H. halimifolium	95	high leverage with high residual Y-variance	unclear
H. halimifolium	103	high X residual	bad spectrum; uneven leaf

3. Supplementary Figures

3.1. Scores plots and RMSEs from PLS models

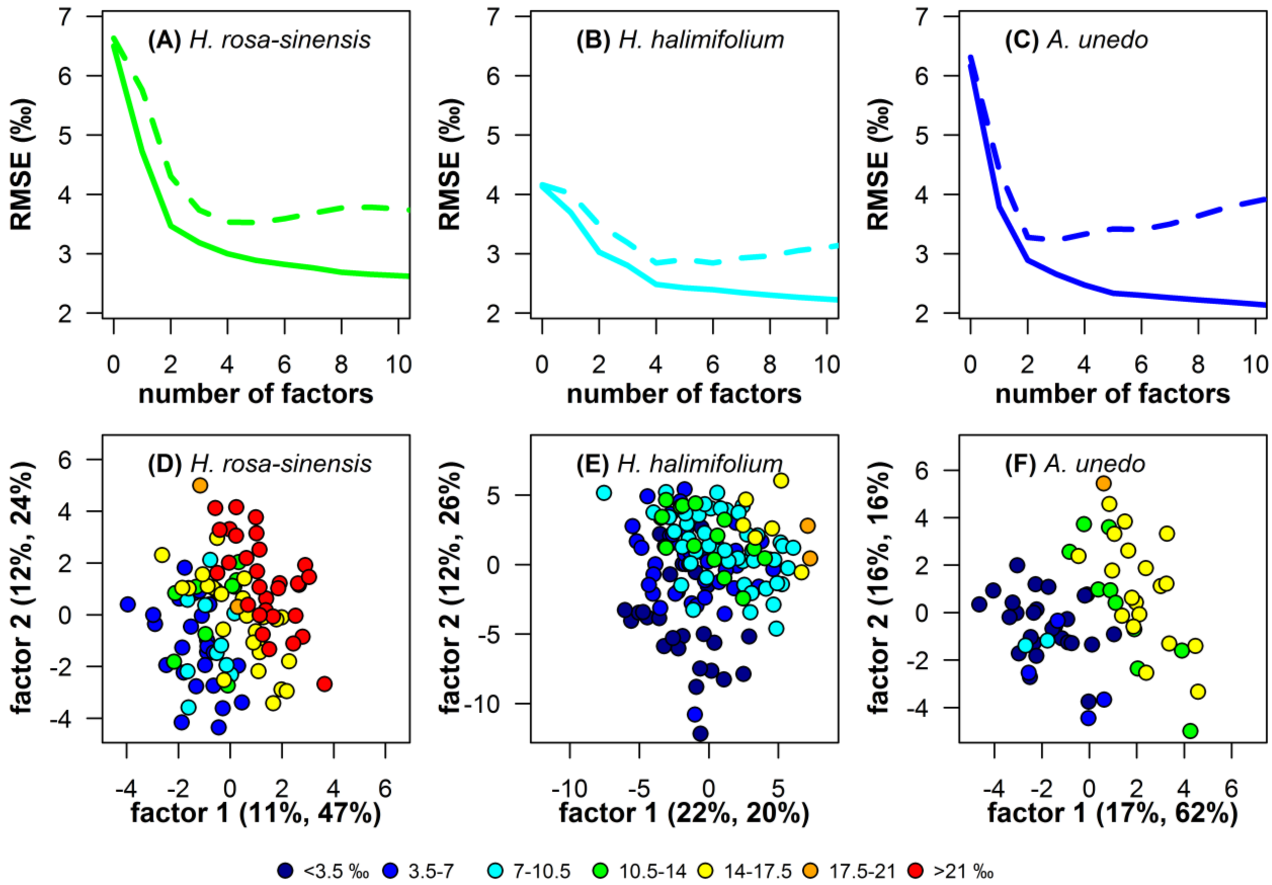

Supplementary Figure S1. Root mean square error (RMSE (‰)) of calibration (solid line) and cross-validation (dashed line) from partial least squares (PLS) regression models plotted against the number of factors (first row) and scores plots of the first two factors (second row) for *Hibiscus rosa-sinensis* (**(A)**, **(D)**), *Halimium halimifolium* (**(B)**, **(E)**) and *Arbutus unedo* (**(C)**, **(F)**). In the scores plots, percentages given in the axes labels are variances in X, Y explained by the respective factor. The color code illustrates $\delta^{15}N$ values of samples.

3.2. Results of the PLS model across all species

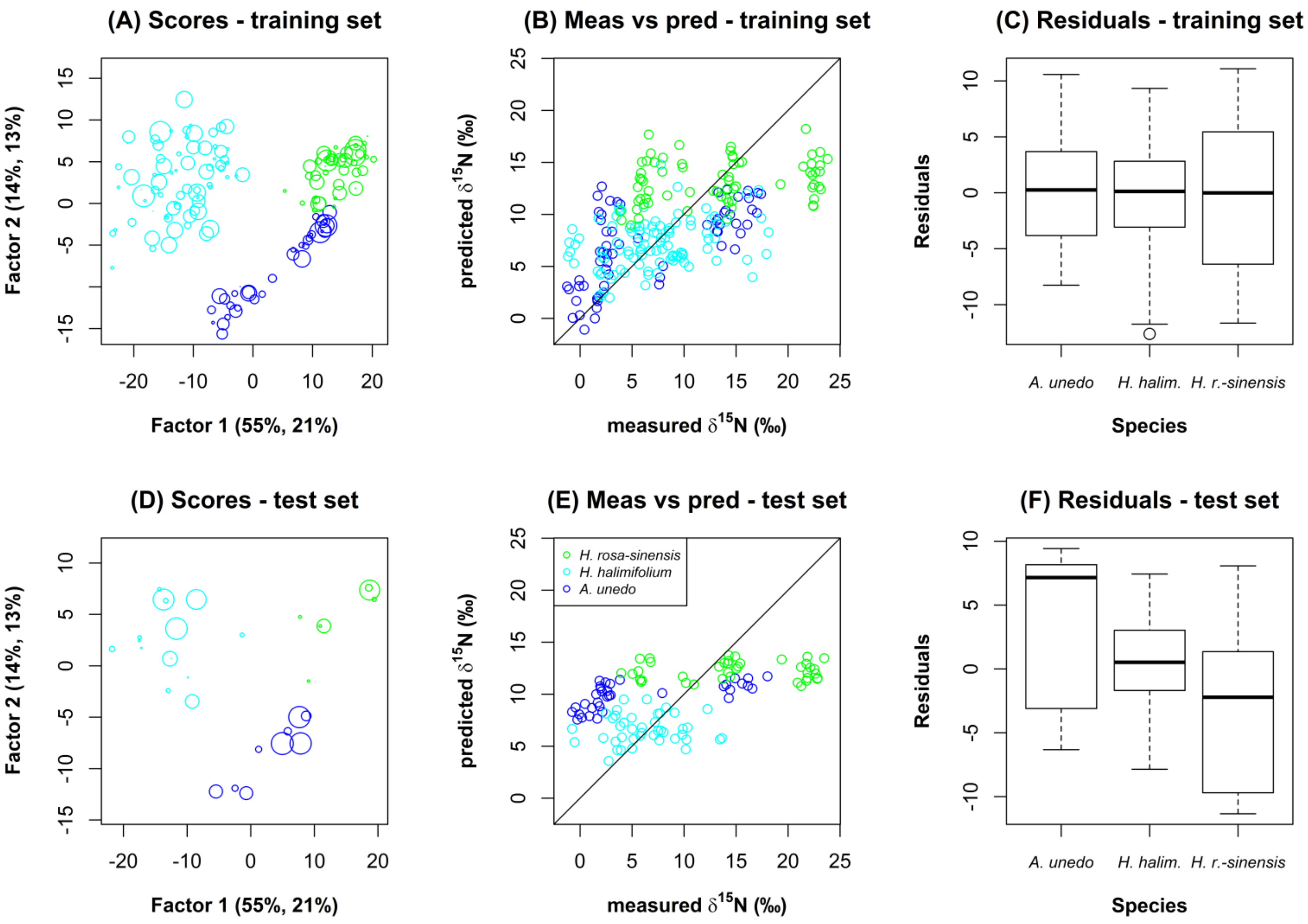

Supplementary Figure S2. Results of a PLS model calculated across all species from the greenhouse experiment, with data divided into training- **(A-C)** and independent test set **(D-F)** by randomly assigning 2/3 of the samples to the training- and 1/3 to the test set. **(A)** and **(D)** show scores plots of the first two factors with X, Y variance explained by the factors given in parenthesis. The size of the bubbles is proportional to the δ^{15}N values; green: *Hibiscus rosa-sinensis*; cyan: *Halimium halimifolium*; blue: *Arbutus unedo*. **(B)** and **(E)** show measured plotted against predicted values (values for cross-validation are shown in **(B)**) and the black line demarcates the 1:1 line. Boxplots in **(C)** and **(F)** show the model residuals grouped by species.
